# Dual 6Pβ-Galactosidase/6Pβ-Glucosidase GH1 Family for Lactose Metabolism in the Probiotic Bacterium *Lactiplantibacillus plantarum* WCFS1

**DOI:** 10.1021/acs.jafc.3c01158

**Published:** 2023-07-06

**Authors:** Laura Plaza-Vinuesa, Ana Sánchez-Arroyo, F. Javier Moreno, Blanca de las Rivas, Rosario Muñoz

**Affiliations:** † Instituto de Ciencia y Tecnología de Alimentos y Nutrición (ICTAN), CSIC, José Antonio Novais 6, 28040 Madrid, Spain; ‡ Instituto de Investigación en Ciencias de la Alimentación (CIAL), CSIC-UAM, CEI (UAM+CSIC), Nicolás Cabrera 9, 28049 Madrid, Spain

**Keywords:** lactose maldigestion, lactose intolerance, glycoside hydrolase, GH1, dual phospho glycosidase

## Abstract

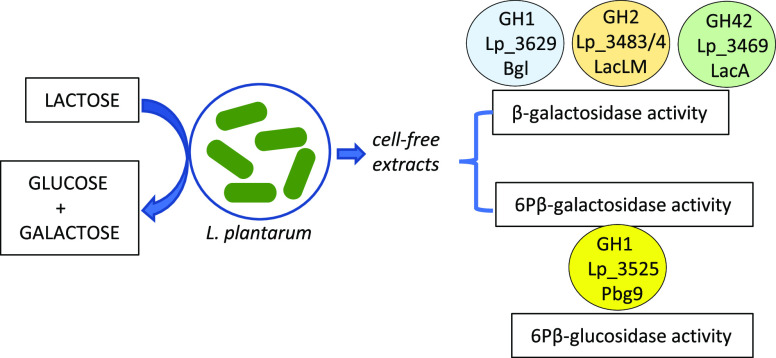

Intestinal lactic acid bacteria can help alleviate lactose maldigestion by promoting lactose hydrolysis in the small intestine. This study shows that protein extracts from probiotic bacterium *Lactiplantibacillus plantarum* WCFS1 possess two metabolic pathways for lactose metabolism, involving β-galactosidase (β-gal) and 6Pβ-galactosidase (6Pβ-gal) activities. As *L. plantarum* WCFS1 genome lacks a putative 6Pβ-gal gene, the 11 GH1 family proteins, in which their 6Pβ-glucosidase (6Pβ-glc) activity was experimentally demonstrated,, were assayed for 6Pβ-gal activity. Among them, only Lp_3525 (Pbg9) also exhibited a high 6Pβ-gal activity. The sequence comparison of this dual 6Pβ-gal/6Pβ-glc GH1 protein to previously described dual GH1 proteins revealed that *L. plantarum* WCFS1 Lp_3525 belonged to a new group of dual 6Pβ-gal/6Pβ-glc GH1 proteins, as it possessed conserved residues and structural motifs mainly present in 6Pβ-glc GH1 proteins. Finally, Lp_3525 exhibited, under intestinal conditions, an adequate 6Pβ-gal activity with possible relevance for lactose maldigestion management.

## Introduction

Lactose is a disaccharide that makes a significant contribution to the nutritive value, flavor, and texture of milk and its derivatives. However, lactose maldigestion and intolerance are the most common disorders of intestinal carbohydrate digestion in humans.^1^ Although virtually all infants can digest milk lactose, there is a slow age-related decline of this metabolic activity, and in fact 75% of adults worldwide are lactose maldigesters.^2^ Ingestion of lactose by a person suffering lactose maldigestion may lead to abdominal bloating and pain, flatulence, nausea, and diarrhea.^1^

Lactose maldigestion refers to a decreased ability to digest lactose due to the lack of β-galactosidase (β-gal) at the brush border membrane of mammalian small intestine-epithelial cells. Individuals who suffer from lactose maldigestion must reduce lactose in their diet and avoid milk consumption. A conventional strategy for lactose maldigestion management is to use milk products with reduced lactose content or to supplement the diet with exogenous β-gal. However, oral administration of β-gal is inconvenient and ineffective because the exogenous enzyme has normally poor pH stability and is rapidly degraded by proteases in the gastrointestinal tract.^3^

β-gal plays an important role in the management of lactose intolerance. Because β-galactosidases are also produced by gut microbiota, intestinal lactic acid bacteria can help alleviate lactose maldigestion. Lactic acid bacteria that can reach and inhabit the intestinal tract may alleviate clinical symptoms brought about by undigested lactose and could promote lactose hydrolysis in the small intestine.^1^ Two different metabolic pathways for the lactose uptake have been reported in lactic acid bacteria. One pathway involves the translocation of intact lactose and further hydrolysis by a β-gal. In the other pathway, lactose is transported into cells via a lactose-specific phosphoenolpyruvate-dependent-phosphotransferase system (PEP–PTS), in which lactose is phosphorylated during transport and then hydrolyzed into 6-phospho-β-d-galactose and d-glucose by 6P-β-galactosidase (6Pβ-gal).^4^

*Lactiplantibacillus plantarum* is one of the few lactic acid bacteria species that has successfully adapted to different foods and is also part of the human colonic microbiota.^5−7^*L. plantarum* possess both metabolic pathways for lactose metabolism, as demonstrated by the fact that cell-free extracts from several strains exhibited β-gal and 6Pβ-gal activities.^8−11^ Several glycosidases exhibiting β-gal activity have been described in *L. plantarum* strains, such as LacA^12,13^ and LacLM.^14−17^ However, the glycosidase(s) responsible for the 6Pβ-gal activity found in *L. plantarum* cell extracts remains unknown.

This work aimed to study 6Pβ-gal activity in the *L. plantarum* WCFS1 strain because it was isolated from human saliva and persists in the digestive tract better than other *Lactobacillus* spp. isolated from the human intestine.^18^ In this study, 6Pβ-gal activity was assayed in 11 glycoside hydrolases from *L. plantarum* WCFS1 possessing 6P-β-glucosidase (6Pβ-glc) activity. One of these proteins exhibited also a high 6Pβ-gal activity and it was subsequently characterized to better elucidate its dual 6Pβ-glc/6Pβ-gal glycosidase activity.

## Materials and Methods

### Bacterial Strains and Growth Conditions

The *L. plantarum* WCFS1 strain used in this study was kindly provided by Dr. Michiel Kleerebezem (NIZO Food Research, The Netherlands). This strain is a colony isolate of *L. plantarum* NCIMB 8826, which was isolated from human saliva. *L. plantarum* WCFS1 was grown in the modified MRS broth (MRS-Gal) at 30 °C. The MRS medium was modified by replacing glucose with galactose to avoid possible catabolite repression by glucose.^19^*Escherichiacoli* DH10B, used for DNA manipulations, and *E. coli* BL21(DE3), used for protein expression, were cultured in the Luria–Bertani (LB) medium containing ampicillin (100 μg/mL) at 37 °C and 140 rpm.

### β-Galactosidase and Phospho-β-Galactosidase Activities in *L. plantarum* WCFS1

Resting cells and cell-free protein extracts from *L. plantarum* WCFS1 were used to determine β-gal and 6Pβ-gal activities by using as substrates synthetic *p*-nitrophenyl-glucoside derivatives, *p*NP-β-d-galactopyranoside (*p*NP-Gal) (Sigma-Aldrich) and *p*NP-6-phospho-β-d-galactopyranoside (*p*NP-6P-Gal) (GoldBio), respectively.

Resting cells and cell-free protein extracts were prepared as previously described.^20^ Briefly, for the resting cell assays, *L. plantarum* WCFS1 cells were cultured in the MRS-Gal medium at 30 °C until 0.5 OD_600nm_ was reached. Then, cells were harvested and washed twice with 0.9% w/v NaCl. After washing, cells were resuspended in 50 mM MOPS buffer (pH 7.0) containing 20 mM NaCl and 1 mM DTT, and the corresponding *p*-nitrophenyl-glycoside (10 mM) was added. Reactions were incubated at 30 °C for 10 min.

For cell-free protein extracts, *L. plantarum* WCFS1 was grown in the MRS-Gal medium at 30 °C, until late exponential phase. Cells were harvested, washed twice with 50 mM MOPS buffer, NaCl 20 mM, 1 mM DTT, pH 7.0, and subsequently resuspended in the same buffer for cell rupture. Bacterial cells were disintegrated by using a French Press (Amicon French pressure cell, SLM Instruments) and subsequently, cells were additionally broken with glass beads in FastPrep TM Fp120 equipment (Savant). The disintegrated cell suspension was centrifuged at 4 °C in order to remove cell debris. Supernatants containing soluble proteins were filtered, and the obtained protein extracts were incubated in the presence of the corresponding substrate (10 mM) at 30 °C for 10 min.

β-gal and 6Pβ-gal activities in resting cells and cell-free protein extracts were determined by a spectrophotometric method previously described.^20^ The rate of hydrolysis of *p*-nitrophenyl (*p*NP) glycosides for 10 min at 30 °C was measured in 50 mM MOPS buffer pH 7.0 containing 20 mM NaCl and 1 mM DTT, at 420 nm in a microplate spectrophotometer PowerWave HT (Bio-Tek, USA). Reactions were stopped by the addition of 1 M sodium carbonate (pH 9.0). Control reactions containing no enzyme were utilized to detect any spontaneous hydrolysis of the substrates tested. Enzyme assays were performed in triplicate.

### Hydrolytic Activity of *L. plantarum* WCFS1 GH1 and GH42 Glycoside Hydrolase Families

Glycoside hydrolases from the GH1 family (Lp_0440, Lp_0906, Lp_1401, Lp_2777, Lp_2778, Lp_3011, Lp_3132, Lp_3512, Lp_3525, Lp_3526, and Lp_3629 proteins) and GH42 family (LacA or Lp_3469) from *L. plantarum* WCFS1 were produced as previously described.^13,20^ The hydrolytic activity of these 12 glycoside hydrolases was determined by using a library of 24 *p*NP-glycoside derivatives.^13^ The assay was performed in a 96-well flat bottom plate (Sarstedt), where each well contained a different substrate (10 mM). Briefly, the reaction consisted of 4 μg protein in 50 mM MOPS buffer pH 7.0 containing 20 mM NaCl and 1 mM DTT. The reaction was incubated at 30 °C for 10 min and stopped by the addition of 1 M sodium carbonate at pH 9.0. Hydrolysis of each *p*NP-glycoside derivative was colorimetrically measured by liberation of *p*-nitrophenolate (*p*NP) at 420 nm in a microplate spectrophotometer PowerWave HT (Bio-Tek, USA). Controls without the enzyme for evaluating spontaneous hydrolysis of the tested substrates were carried out. Experiments were performed in triplicate, and results were expressed as an average activities ± standard deviation.

### Temperature and pH Effects on Lp_3525 6Pβ-glu and 6Pβ-gal Activities

The effects of temperature and pH on Lp_3525 were determined using *p*NP-6-phospho-β-d-glucopyranoside (*p*NP-6P-Glc) and *p*NP-6-phospho-β-d-galactopyranoside (*p*NP-6P-Gal) as substrates, for 6Pβ-glc and 6Pβ-gal activities, respectively. The optimal pH was determined by using citrate (pH 3.0), acetic acid-sodium acetate (pH 4.0–6.0), MOPS (pH 6.5 and 7.0), and Tris–HCl (pH 8.0) buffers (50 mM). The optimal temperature was assayed by incubation of Lp_3525 in 50 mM MOPS buffer pH 7.0 containing 20 mM NaCl and 1 mM DTT at 4, 22, 30, 37, 45, and 65 °C. Assays were performed in triplicate.

## Results and Discussion

### Identification of the Proteins Involved in Lactose Hydrolysis in *L. plantarum* WCFS1

Two different pathways for lactose hydrolysis have been described in lactic acid bacteria, involving proteins exhibiting β-gal and 6Pβ-gal activities. These two activities were reported in crude cell-free extracts from *L. plantarum* OSU in a survey of lactobacilli for β-galactosidase and phospho-β-galactosidase activities using *p*NP-galactoside derivatives as substrates.^8^ Later, using the same substrates, both activities were also identified in additional *L. plantarum* strains, extracts,^10^ or toluene-treated cell suspensions.^11^ However, none of these assays evaluated the activity exhibited by *L. plantarum* whole cells. When the lactose uptake occurs through the PEP-PTS, whole cells should not exhibit 6Pβ-gal activity because lactose needs to be phosphorylated during its transport into the cell. In order to know whether β-gal and 6Pβ-gal activities exist in the probiotic *L. plantarum* WCFS1 strain, both activities were assayed in resting cells and in cell-free protein extracts by using *p*NP-Gal and *p*NP-6P-Gal as substrates. As expected, resting cells did not show 6Pβ-gal activity, and they exhibited only a low β-gal activity (0.47 ± 0.14 μmol min^–1^ mg^–1^). By contrast, both activities were observed at the same extent in cell-free extracts, β-gal (3.67 ± 0.08 μmol min^–1^ mg^–1^) and 6Pβ-gal activity (3.69 ± 0.06 μmol min^–1^ mg^–1^). This is the first report describing similar β-gal and 6Pβ-gal activities in *L. plantarum* cell-free extracts. Previously, higher β-gal than 6Pβ-gal activities were reported in *L. plantarum* strains,^8,10,11^ except for *L. plantarum* ATCC 14917 and *L. plantarum* ATCC 8014 strains, which exhibited higher 6Pβ-gal activity.^11^ As β-gal activity was generally the predominant activity in the previous reports, it was speculated that 6Pβ-gal could be a spurious enzymatic activity in *L. plantarum*.^15^

Once the presence of β-gal and 6Pβ-gal activities in *L. plantarum* WCFS1 cell-free extracts was clearly confirmed, the proteins involved in both activities should be identified. The available complete genome sequence of *L. plantarum* WCFS1 revealed the existence of a high number of proteins annotated as “glycoside hydrolases”. Among the families included in the carbohydrate-active enzyme (CAZy) database that contain proteins possessing β-gal activity, only GH1, GH2, and GH42 families are present in *L. plantarum* WCFS1. The presence of proteins annotated as β-gal belonging to the families GH1 (Lp_3629), GH2 (Lp_3483 and Lp_3484), and GH42 (Lp_3469) are found in the genome of *L. plantarum* WCFS1. *L. plantarum* β-gal from GH2 (LacLM) and GH42 (LacA) families have been previously recombinantly produced and biochemically characterized.^12,13,15,16^ Both β-gal possess high transgalactosylation activity for the synthesis of prebiotic galacto-oligosaccharides or other galactosylated derivatives.^12,13,16^ In relation to GH1 family β-gal, it was experimentally proven that Lp_3629 possesses β-gal activity, and not β-glc activity as it was originally annotated.^21^ When immobilized, this β-gal successfully catalyzed the hydrolysis of lactose and lactulose, and the formation of different oligosaccharides from galactose and lactulose was detected.^22^

According to the CAZy database, 6Pβ-gal is categorized as a GH1 family enzyme. A total of 21 different enzymatic activities are currently classified in the GH1 family. Some enzymes, however, have broad substrate specificity, meaning that they can utilize more than one substrate and catalyze more than one reaction. The complete genome of *L. plantarum* WCFS1 possesses 11 genes encoding for GH1 family proteins that are annotated as 6PB-glc; however, there is no protein annotated as putative 6Pβ-gal. These 11 GH1 proteins have been recently characterized. All of them presented 6Pβ-glc activity, and in addition, 8 proteins exhibited 6-phospho-β-d-thioglucosidase activity.^20^ Therefore, it is possible that some of these GH1 proteins could also possess 6Pβ-gal activity, being responsible for the 6Pβ-gal activity exhibited by *L. plantarum* WCFS1 cell-free extracts.

The 11 GH1 6Pβ-glc proteins and LacA β-gal were recombinantly produced as previously described.^13,20^ The hydrolytic activity of these enzymes was assayed by using *p*NP-β-d-galactopyranoside (*p*NP-Gal) and *p*NP-6-phospho-β-d-galactopyranoside (*p*NP-6P-Gal) as substrates, for β-gal and 6Pβ-gal activities, respectively. LacA GH42 proteins were used as the control for β-gal activity.^13^ In addition, the catalytic activity of these proteins toward 24 *p*NP glucoside derivatives was also assayed. Despite all the GH1 proteins exhibiting 6Pβ-glc activity,^20^ only one of them, Lp_3525 (Pbg9), effectively hydrolyzed *p*NP-6P-Gal and showed high 6Pβ-gal activity (Table 1). Low 6Pβ-gal activity was observed in Lp_1401, Lp_2777, and Lp_2778 proteins. As shown in Table 1, 8 out of 11 GH1 proteins also presented detectable β-gal activity, even though their activity levels were 13 to 35-fold lower than that exhibited by LacA, a GH42 β-gal (Table 1).^13^ None of these GH1 proteins showed activity against β-glc or other *p*NP-derivatives assayed. Surprisingly, LacA β-gal exhibited high hydrolytic activity on *p*NP-α-l-arabinopyranoside and *p*NP-β-d-fucopyranoside (data not shown).

**Table 1:** Activity of Glycoside Hydrolases from *L. plantarum* WCFS1 against *p*NP-β-Gal Derivatives (μmol Min^–1^ Mg^–1^)^a^

		activity	
CAZy family	protein	β-Gal	6Pβ-Gal
GH42	Lp_3469	3.1 ± 4.0 × 10^–2^	ND
			
GH1	Lp_0440	0.10 ± 5.0 × 10^–3^	ND
	Lp_0906	0.23 ± 2.0 × 10^–2^	ND
	Lp_1401	0.11 ± 1.0 × 10^–2^	0.10 ± 1.0 × 10^–2^
	Lp_2777	ND	0.27 ± 1.0 × 10^–2^
	Lp_2778	0.09 ± 5.0 × 10^–2^	0.17 ± 5.0 × 10^–3^
	Lp_3011	0.09 ± 4.0 × 10^–3^	ND
	Lp_3132	0.10 ± 4.0 × 10^–3^	ND
	Lp_3512	0.13 ± 8.0 × 10^–3^	ND
	Lp_3525	ND	1.14 ± 4.0 × 10^–2^
	Lp_3526	ND	ND
	Lp_3629	0.09 ± 4.0 × 10^–3^	ND

a ND, not detected.

The obtained results indicated that Lp_3525 has broad specificity as it could hydrolyze more than one phosphorylated substrate.

### Sequence Analysis of the Lp_3525 “Dual” 6Pβ-gal/6Pβ-glc from *L. plantarum* WCFS1

Comparative structural analysis suggests that a tryptophan instead of a methionine or alanine residue at subsite −1 may contribute to the catalytic and substrate selectivity to structurally similar 6Pβ-gal and 6Pβ-glc assigned to the GH1 family.^23^ Galacto- and gluco-derived substrates are very similar and have identical structures, differing only in the position of their O4 hydroxyl group, which is an axial position in the galacto-epimer versus an equatorial position in the gluco-epimer. It was hypothesized that both enzymes evolved from a common ancestor, but at some point, 6Pβ-gal evolved independently, so that a tryptophan residue was acquired in order to accommodate galactose-6′P (rather than glucose 6-phosphate) at the active site of subsite −1.^23^

**Figure 1:**
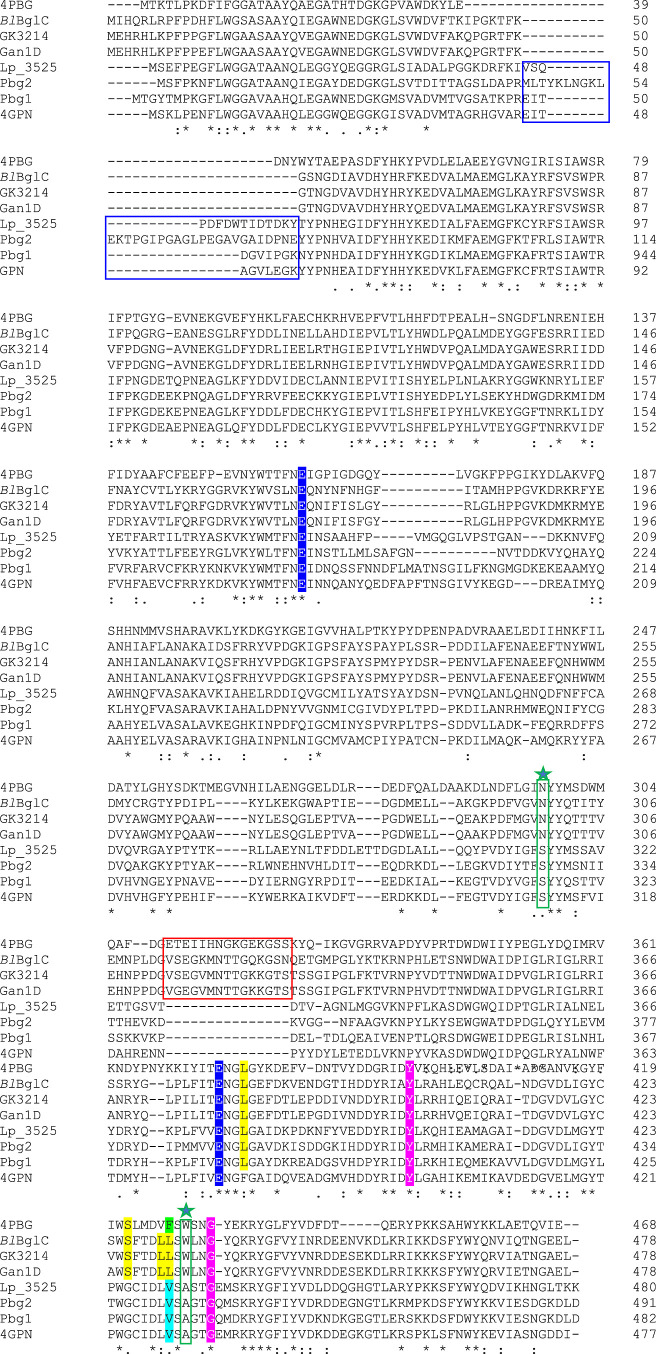
Comparison of amino acid sequences of GH1 family proteins possessing 6Pβ-gal, 6Pβ-glc, and dual 6Pβ-gal/6Pβ-glc activity. 4PBG, LacG from *L. lactis* subsp. *lactis* (P11546) and 4GPN from *S. mutans* ATCC 700610 (Q8DT00) possessed PDB structures and were previously used as models for 6Pβ-gal and 6Pβ-glc GH1 proteins, respectively.^27^ The alignment includes Lp_3525 from *L. plantarum* WCFS1 (CCC80491), Pbg1 (BAA20086) and Pbg2 (BAA25004) from *L. gasseri*, and proteins described to possess dual 6Pβ-gal/6Pβ-glc activity, such as *Bl*BglC from *B. licheniformis* (UPI000043D040), GK3214 from *G. kaustophilus* HTA426 (Q5KUY7), and Gan1D from *Geobacillus stearothermophilus* (W8QF82). Residues that are identical (*), conserved (:), or semiconserved (·) in all sequences are indicated. Dashes indicate gaps introduced to maximize similarities. The conserved catalytic acid/base and nucleophilic Glu residues are marked in white letters and highlighted in navy blue. Residues conserved in the particular activity in which it belongs are highlighted in yellow for dual 6Pβ-gal/6Pβ-glc, green for 6Pβ-gal, and blue for 6Pβ-glc.^27^ Residues described as specific for one activity but conserved in the three activities studied are marked in white letters and highlighted in pink.^27^ The residues Asn/Ser and Trp/Ala which defined the preference for 6P-β-Gal or 6P-β-Glc substrates^29^ are marked in green boxes and with a green star. The long extra C-terminal segment present in 6Pβ-glc is marked in a blue box. The conserved lid motif present in 6Pβ-gal that blocks the entrance to the active site is marked in a red box.

Currently, a series of GH1 enzymes with dual 6Pβ-gal/6Pβ-glc activity has been described; although, only the complete 3D crystal structure of Gan1D from *Geobacillus stearothermophilus* is known.^24,25^ Gan1D has been shown to exhibit bifunctional activity possessing both 6Pβ-gal and 6Pβ-glc activities. The different ligands trapped in the active site adopt different binding modes to the protein, providing a structural basis for the dual gal/glc activity observed for Gan1D enzymes. In this protein, specific mutations were performed on one of the active site residues (Trp-433), shifting the enzyme specificity from dual activity to a significant preference toward 6Pβ-glc activity (Figure 1).^25^ When Trp-433 was mutated to either Ala or Met, both mutant enzymes were more active toward glucose substrates in comparison to galactose substrates.^26^ It was previously described that this tryptophan residue plays a functional role in the differentiation of the catalytic properties of 6Pβ-gal and those of 6Pβ-glc assigned to the GH1 glycoside hydrolase family.^23^ This residue is also present in GK3214, a thermostable 6Pβ-glycoside GH1 family from *Geobacillus kaustophilus* HTA426,^27^ which hydrolyzed 6Pβ-gal and 6Pβ-glc with high activity. Recently, Veldman et al.^28^ described the differences between gluco and galacto substrate-binding interactions in a dual 6Pβ-gal/6Pβ-glc GH1 enzyme, *Bl*BglC, from *Bacillus licheniformis*. Moreover, they described the similarities and differences in active-site residue interactions between dual 6Pβ-gal/6Pβ-glc, 6Pβ-gal, and 6Pβ-glc activities, taking *Bl*BglC from *B. licheniformis*, 4PBG (LacG) from *Lactococcus lactis* subsp. *lactis*, and 4GPN (Bgl) from *Streptococcus mutans* as protein models for each specific activity, respectively (Figure 1). Among the residues conserved in the dual 6Pβ-gal/6Pβ-glc activity, Ser-426 and Leu-431 (*Bl*BglC numbering) involved in the hydrogen bond are not conserved in Lp_3525. Instead, Gly-426 and Val-431 residues (Lp_3525 numbering) are found. These residues are conserved in the 6Pβ-glc 4GPN from *S. mutans* (Figure 1), and, moreover, the Val residue is described as a residue conserved in all the 6Pβ-glc.^28^ The presence in Lp_3525 of typical residues found in 6Pβ-glc and the absence of the tryptophan residue present in 6Pβ-gal (Trp-429 in 4PBG) or in the previously described dual 6Pβ-gal/6Pβ-glc protein (Trp-433 in *Bl*BglC) suggested that Lp_3525 belongs to a different dual 6Pβ-gal/6Pβ-glc protein group (Figure 1).

The sequence of Lp_3525 has typical residues conserved in 6Pβ-glc.^20^ Thus, the catalytic acid/base and nucleophilic Glu residues (Glu-181 and Glu-378), the residues involved in glycon binding (−1 subsite), and those involved in aglycon binding (+1 subsite) are conserved.^20^ Moreover, the Lp_3525 sequence presents a long extra C-terminal segment unique to 6Pβ-glc, which varies in length and sequence. This is a typical 6Pβ-glc motif that is absent in the 6Pβ-gal hydrolases.^29^ In addition, Lp_3525 lacks the lid motif present in 6Pβ-gal that blocks the entrance to the active site (Figure 1).^29^ Lp_3525 contains the residues Ser-315 and Ala-433, which have been described in 6Pβ-glc (Ser-311 and Ala-431 in 4GPN) instead of the asparagine and tryptophan present in the 6Pβ-gal (Asn-297 and Trp-429 in 4PBG) (Figure 1).^30^ All these sequence features are supported by the sequence identity exhibited by Lp_3525, which is more similar to 4GPN 6Pβ-glc (52.88% identity) than to 4PBG 6Pβ-gal (35.76% identity). All these data seem to indicate that Lp_3525 belongs to a dual 6Pβ-gal/6Pβ-glc enzyme group but more closely resembling the structural features of 6Pβ-glc proteins.

However, *Bl*BglC, GK3214, and Gan1D proteins have been previously described as “dual 6Pβ-gal/6Pβ-glc”,^25,27,28^ despite presenting a similar sequence identity to 4GPN and 4PBG (35.16 and 40.75% identity, respectively). Moreover, they possess residues and motifs specific for 6Pβ-gal, such as the asparagine and tryptophan residues (Asn-297 and Trp-429 in 4PBG),^25,30^ and the lid motif that blocks the entrance to the active site.^29^ Additionally, the long extra C-terminal segment typical of 6Pβ-glc is absent in BlBglC, GK3214, and Gan1D proteins. These data indicated, that contrarily to Lp_3525, these dual 6Pβ-gal/6Pβ-glc are more similar to 6Pβ-gal enzymes.

In order to know if Lp_3525 is the only dual 6Pβ-gal/6Pβ-glc protein described so far that shares structural features with 6Pβ-glc proteins, the activity of the reported GH1 proteins was revised. In *Lactobacillus gasseri* ATCC33323, four enzymes showing 6Pβ-gal activity (LacG1, LacG2, Pbg1, and Pbg2) were described. Phylogenetic analysis showed that LacG1 and LacG2 belongs to the 6Pβ-gal cluster and Pbg1 and Pbg2 belongs to the 6Pβ-glc cluster, as they showed closer similarity to 6Pβ-glc (about 50%) than to 6Pβ-gal (30–35%).^31^ The four *L. gasseri* GH1 glycosidases exhibited dual 6Pβ-gal/6Pβ-glc activity, as they were able to hydrolyze *o*NP-6P-Gal and *o*NP-6P-Gal.^32^ As in the case of Lp_3525, Pbg1 and Pbg2 (50.74 and 52.59% identical to Lp_3525, respectively) showed all the residues and motifs conserved in 6Pβ-glc (Figure 1).

Therefore, so far, the differences in gluco and galacto substrate-binding interactions in dual 6Pβ-gal/6Pβ-glc GH1 glycosidases have only been analyzed in proteins sharing sequence and structural features with 6Pβ-gal (such as *Bl*BglC, GK3214, and Gan1D).^28^ However, gathered evidence indicates that there is a different group of GH1 glycosidases possessing dual 6Pβ-gal/6Pβ-glc activity whose the structural basis for their enzyme bifunctionality remains unknown.

### 6Pβ-gal and 6Pβ-glc Activity Properties of Lp_3525 from *L. plantarum* WCFS1

Some studies regarding dual 6Pβ-gal/6Pβ-glc GH1 glycosidases have been focused on the elucidation of the gluco- and galacto-binding interactions and did not include the study of their biochemical properties. The optimum temperature and pH were studied in LacG1 and LacG2 from *L. gasseri* ATCC 33323^T^.^32^ Both proteins exhibited dual 6Pβ-gal/6Pβ-glc activity and similarly to *Bl*BglC, GK3214, and Gan1D glycosidases belonged to the 6Pβ-gal group. Despite these proteins showing an identical optimal temperature for both substrates, they possessed different optimal pH for each activity.^31^ The optimum pH of LacG1 was 6.0 for *o*NP-6P-Gal and 7.0 for *o*NP-6P-Glc, and LacG2 showed optimum pH at 5.5 for *o*NP-6P-Gal and 6.0 for *o*NP-6P-Glc.^32^

**Figure 2:**
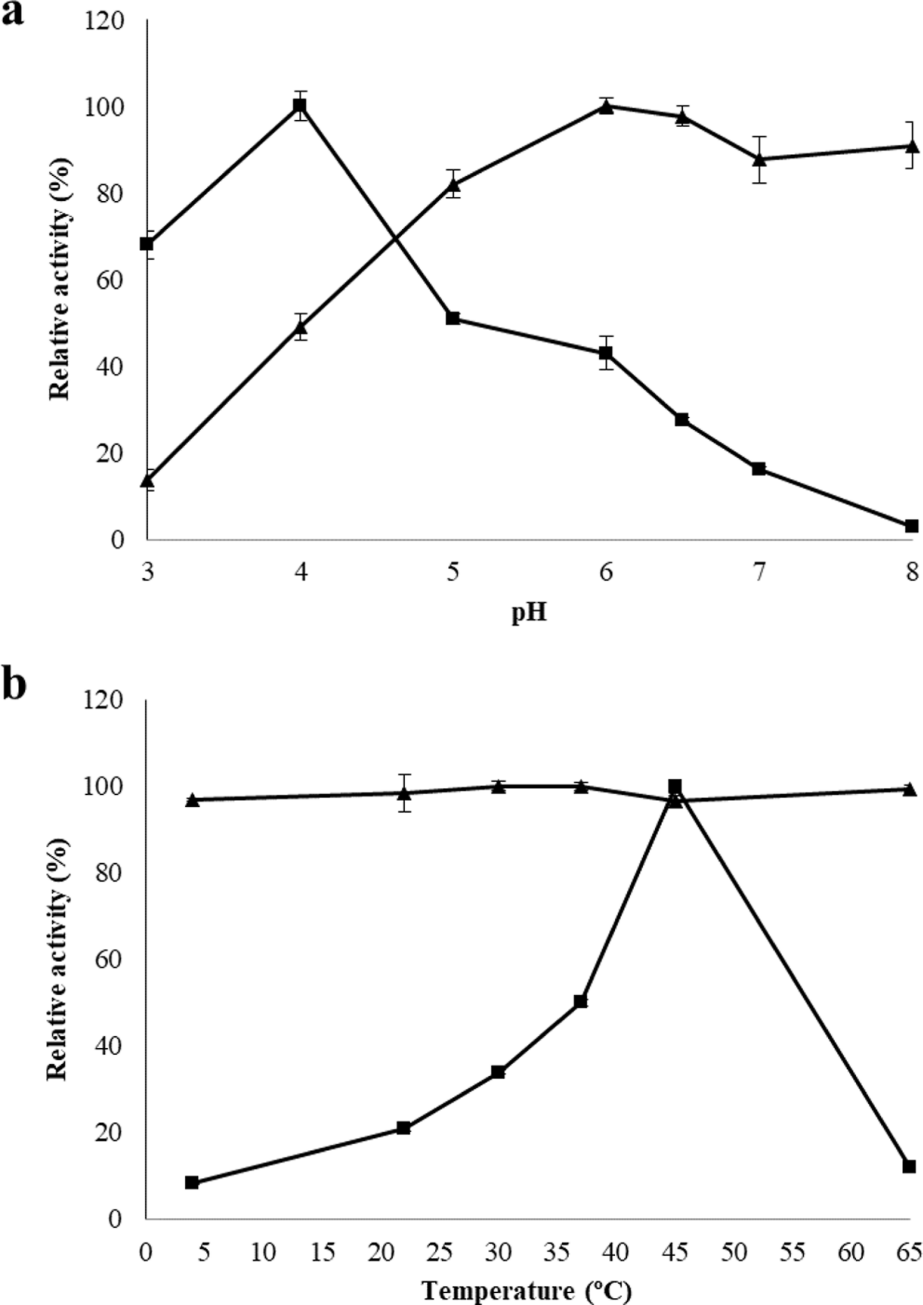
Temperature and pH effects on Lp_3525 activities from *L. plantarum* WCFS1. (a) Relative activity of Lp_3525 versus pH by using *p*NP-6P-Gal (filled square) or *p*NP-6P-Glc (filled triangle) as substrates. (b) Relative activity of Lp_3525 versus temperature by using *p*NP-6P-Gal (filled square) or *p*NP-6P-Glc (filled triangle) as substrates in MOPS buffer (50 mM, pH 7) containing 20 mm NaCl and 1 mm DTT. The experiments were done in triplicate. Mean values and standard errors are shown. The observed maximum activity was defined as 100%.

In this work, a similar study was performed for Lp_3525, the dual 6Pβ-gal/6Pβ-glc from *L. plantarum* WCFS1 belongs to the 6Pβ-glc group. Contrarily to LacG1 and LacG2 from *L. gasseri* ATCC 33323^T^, Lp_3525 showed different behaviors in relation to the temperature depending on the substrate used. When using *p*NP-6P-Gal as the substrate, Lp_3525 showed a clear optimal temperature at 45 °C; however, it exhibited similar maximal activity at all the temperatures (from 4 to 65 °C) assayed with *p*NP-6P-Glc (Figure 2b). The Lp_3525 optimal temperature for *p*NP-6P-Gal is similar to that described for LacG1 (40 °C) and LacG2 (40 °C) from *L. gasseri* for both substrates.^32^ In addition, Lp_3525 possessed a different optimal pH for each enzymatic activities, being more acidic for 6Pβ-gal (pH 4.0) than for 6Pβ-glc (pH 6) activity (Figure 2a). This behavior was also observed for LacG1 and LacG2 from *L. gasseri*, on which the optimal pH was more acidic when *p*NP-6P-Gal was used as a substrate than when *p*NP-6P-Glc was used.^32^

In relation to 6Pβ-gal activity, acidic optimal pH was observed in several 6Pβ-gal previously described. Similar to Lp_3525, CelB 6Pβ-gal from *Pyrococcus furiosus* exhibited 4.0 as the optimal pH.^33^ 6Pβ-gal from *Lactobacillus casei*(8) and Pbp1 and Pbg2 from *L. gasseri* JCM 1031^34^ presented 5.0–5.5 optimal pH values. However, enzyme activity was nearly constant over a broad pH range from 5.0 to 8.0 in LacG 6Pβ-gal from *Streptococcus cremoris*.^35^ Optimal temperature for 6Pβ-gal hydrolases varies from 30 °C (Pbg2 from *L. gasseri* JCM 1031)^34^ to 50 °C (LacG from *S. cremoris* and Pbg1 from *L. gasseri* JCM1032).^34,35^ Most of these 6Pβ-gal were inactivated above 60 °C, as it happened in Lp_3525 whose optimal temperature was 45 °C (Figure 2b). Compared to the 6Pβ-gal hydrolases previously described, it seems that Lp_3525 possesses optimal temperature and pH values similar to Pbg1 from *L. gasseri* JCM1031, which is also a dual 6Pβ-gal/6Pβ-glc GH1 enzyme belonging to the 6Pβ-gal group (Figure 1).

When Lp_3525 6Pβ-glc activity was compared to other 6Pβ-glc previously described, it was observed that Lp_3525 possessed a broad range of optimal temperature and pH (Figure 2). Lp_3525 showed maximal activity at all the temperatures assayed, from 4 to 65 °C. BglD and CelD 6Pβ-glc from *Oenococcus oeni* also showed high activity at low temperatures because they retained at 4 °C more than 70% of their maximal activity; however, their activity was reduced to 21% at 50 °C (BglD)^36^ or 2% at 60 °C (CelD)^37^ whereas Lp_3525 retained at 65 °C more than 95% of its maximal activity (Figure 2b). As mentioned above, optimal pH for Lp_3525 6Pβ-glc activity is less acidic (6.0) than for 6Pβ-gal activity (4.0). A similar optimal pH was reported for 6Pβ-glc previously described, such as Pbgl25-217 being isolated from a metagenome from black liquor sediments,^38^ Spy1599 from *Streptococcus pyogenes*,^39^ or LacG1 and LacG2 from *L. gasseri* ATCC 33323.^32^ It is interesting to note that Lp_3525 retained more than 90% of 6Pβ-glc activity at pH 8.0, whereas 6Pβ-glc from *O. oeni* only retained 12% maximal activity at pH 7.0 (BglD)^36^ or 25% at pH 7.5 (CelD).^37^

The obtained data confirmed that similarly to dual 6Pβ-gal/6Pβ-glc belonging to the 6Pβ-gal group (such as LacG1 and LacG2 from *L. gasseri* ATCC 33323), Lp_3525, which belongs to the 6Pβ-glc group, possessed different biochemical properties concerning the hydrolyzed substrate, *p*NP-6P-Gal or *p*NP-6P-Glc. Moreover, Lp_3525 exhibited a broad range of temperatures and pH for its maximal 6Pβ-glc activity, although it displayed more constricting conditions for 6Pβ-gal activity.

*L. plantarum* is one of the few lactic acid bacteria species in the human intestine,^40,41^ and some strains have been used as probiotics; therefore, it is important to elucidate their ability for lactose metabolism. Although *L. plantarum* strains possessed both 6Pβ-glc and 6Pβ-gal activities, sequence analysis indicates that the *L. plantarum* WCFS1 genome possesses 11 genes annotated as 6Pβ-glc, whereas no putative 6Pβ-gal was found to metabolize lactose. In lactic acid bacteria, a strong relationship between 6Pβ-gal and 6Pβ-glc exists in the lactose metabolism.^4^ For example, a *lac*G-deficient strain of *L. lactis* grew slowly in a lactose medium using 6Pβ-glc for lactose degradation.^42^ It has been described in *L. gasseri* ATCC 33323 that lactose induced not only 6Pβ-gal (lactose-specific) but also 6Pβ-glc. This strain contained seven different genes associated with 6Pβ-glycosidases, at least four of them encoded dual 6Pβ-gal/6Pβ-glc proteins. Pbg1 and Pbg2 belong to the 6Pβ-glc group described in this study for the first time, whereas the primary enzymes for lactose utilization, LacG1 and LacG2, belong to the 6Pβ-gal group previously described.^31,32^ In *L. plantarum* WCFS1, among the 11 proteins having 6Pβ-glc activity,^20^ only Lp_3525 possessed high 6Pβ-gal activity. Although it appears to be a waste of energy that several enzymes possess apparently the same function, results previously obtained by using 6Pβ-glc mutants,^43^ and those described in this work confirmed that not all the proteins are functionally complementary. It has been described that the gene encoding Lp_3525, the *L. plantarum* WCFS1 dual 6Pβ-gal/6Pβ-glc protein, is controlled by carbon catabolite repression,^44^ but further studies are needed in order to know the regulation and expression of Lp_3525 in the presence of lactose and different carbon sources, and its possible relevance for lactose maldigestion management.

In addition to lactose metabolism, in this work, we have demonstrated that Lp_3525 could be able to hydrolyze phospho-β-galacto/gluco-derived substrates over a broad temperature and pH ranges, especially for the 6Pβ-glc activity. Although the capability of phospho-β-glycosidase activities is still rather unexplored, this type of enzymes may catalyze the degradation of phosphorylated glucosides, glycosylated lignin, and fiber-related disaccharides (e.g., cellobiose and gentiobiose) during plant-based fermentations and contribute to the release of a wide range of phenolic compounds.^45^ As a consequence, these enzymes could be useful in bioprocessing approaches for the valorization of some cereal byproducts following their inclusion in lactic acid bacteria starters with targeted metabolic activities.^46^
